# The Keeping of Personal Records

**Published:** 1920-04-24

**Authors:** 


### MEDICAL DOMESDAY BOOK.

### The Keeping of Personal Records.

It has been suggested that the records now being made by those in charge of maternity centres should be passed on to the local medical authorities, where each would become a record of the individual during the school period. Hence they would pass to the medical practitioner who attended the patient during adult life, and ultimately go to the Ministry of Public Health. The idea is an attractive one, which might have far-reaching results to the individual patients, to the furtherance of our knowledge, and to the members of our profession. It is further suggested that such a record, if carefully kept by a series of good observers over a number of years, would be of considerable advantage to most of us when taken ill; that it might often help in the diagnosis of our case, and make prognosis more easy and more accurate. As a result the correct line of treatment would be embarked upon in an early stage of our illness, and valuable warnings not to neglect a minor ailment would occasionally be afforded. On the other hand, such a record cannot be without its dangers; it might tend to make many of us more introspective; and we should see many running round to the doctor with some trivial complaint because they felt that it must be recorded on their sheets. We can foresee in this way an increase of functional disease, and such a classifying as organic of many minor functional disturbances that the disadvantage from this would outweigh the occasional more early diagnosis of an organic lesion. Functional disease is one of the banes of our times, and anything which affords a possibility of its increase must rigorously be inquired into before it is adopted.

### The Furtherance of Medical Knowledge.

If a number of such careful records were preserved we should have a most valuable means of gaining knowledge. We are not great believers in the value of statistics; we regret that they are more often twisted to fit into one theory or another than used as a foundation upon which new ideas can be built. But we must admit that such disadvantages frequently result from an insufficiency of data. We see deductions in percentages made on fifty or sixty cases; whereas if millions of records were kept data might be obtained of several hundred strictly analogous cases from which deductions could more reasonably be made as to the value of some obscure physical sign or the importance of some unusual symptom. It is true that the compilation of such statistics would entail an enormous amount of labour; it would necessitate the wisest critical powers to decide which should be included in a given list, and the most rigorous self-control to exclude any case which was not strictly analogous to the series under consideration. Such a labour would be expensive and must be added to the expense of storing, sorting, and indexing which would be necessary if the facts acquired were to be rendered valuable.

To the medical practitioner we feel that it would mean but little more than a considerable increase of work, and of a kind of work which is felt by many to be drudgery of the dullest kind, and for which many are totally unsuited. The records would occasionally help in diagnosis; but the sheets which had to be passed from one practitioner to another with each change of address of the patient, or each variation of decision as to who should attend him, might become a source of great anxiety to the doctor if he had honestly put down what he had found or what he had thought. Unless the sheets were recognised as being privileged we can imagine many suits at law arising out of them. Unless they were kept very strictly confidential we are sure that many a black look would be levelled at a doctor, who might well wonder why he had recently lost as a friend someone who years ago had been a patient. If we honestly, carefully, and accurately kept a record of every patient whom we saw it would greatly improve our work. We know of nothing more conducive to thoroughness and care of examination than the necessity of putting down in writing what has been observed. It makes one's conscience uncomfortably active to realise how many points have been missed, how many inaccurately noted, and how many more described in a slip-shod manner. We have no doubt that the whole status of the profession would be raised as a result of increase of knowledge, increase of accuracy, and increase of care if such a scheme were honestly and thoroughly carried out.

### The Big "Ifs."

It may be noted that in the above summary the disadvantages of the scheme have been seen to be dependent upon factors that would very definitely arise, but the advantages are all conditional upon the records being kept, upon their being kept accurately, and upon the accuracy being complete. That these are very big "Ifs" will be realised when we consider in the light of experience the value of records which are at present being kept.

Those who have been registrars at a big hospital to which a teaching school is attached know the amount of energy that has to be put into the post in order to get the reports or notes written by the student at all. Here and there are a few men who will settle down at once and really try to write a report, and among these an occasional student seems to know at once what is vital to the report and what is not. But in the majority of cases it is a continual drive to get in the reports, and when they are written much of what is put down is of little or no value. When he turns to the bound reports of former years he finds that they are not of the value he would hope them to be. Even the terminology of former years varies from that of to-day to such an extent that it is hard to tell what were the opinions of those who saw the case fifteen or twenty years ago.

### Recent Military Experience.

It may be suggested that the above criticisms on hospital medical and surgical reports are due to their being written by students who do not know the essentials of medicine and have not yet developed a sense of responsibility. We fear that our recent Army experience proves that notes are not written a bit better by the average qualified man or woman than they are by the student—worse, in fact, in just such a proportion as control is removed from over them. The use of the field medical card will explain our point. In its last form it was so drawn up that the minimum effort was needed so that points which were thought essential could be entered. Those in the forward area used a considerable pressure in order to ensure that notes should be entered on the card by the various M.O.s through whose hands the patient passed. Under the conditions of active service the sick man is not the patient of an individual; he passes through

**Medical Domesday Book—(*continued*).**

the hands of a succession, being under one man for forty-eight hours, perhaps, before he is passed on. Under such circumstances any notes are of the utmost value in the diagnosis and treatment of the case. In spite of all this the M.O. at the base was continually at a loss with regard to the cases that came along, owing to the absolute insufficiency or total absence of notes upon the field medical card.

Again, it may be suggested that M.O.s could not be expected to write notes in the front area during the stress of an action or an epidemic. To this the answer is that the average of notes on field medical cards was just as bad during the quiet times as it was during the strenuous ones; that medical units at certain places on the lines of communication or at the base were notoriously bad for the ways in which the field medical cards were filled up, and that though the average was bad, this work from certain units and by certain individuals was good even in the hardest times. If any further evidence were needed it is that the notes kept by the M.O.s at the base were as bad as any that came down the line on the field medical card. A stiff card printed in pink—we believe it belongs to the " W " series of Army Forms—has been introduced, upon which the M.O. writes his notes. The experience of taking over wards or tents from other medical officers at base hospitals would give anyone the poorest idea of the value of these notes. Rumour had it that their ultimate destination was some Government Department lodged temporarily in the British Museum, and that the medical statistics of the war would ultimately be made up from them. But the thing that would convince anyone of the total impossibility of getting any body of medical men and women to write reasonable notes is an Army Form known as the B 179. On this the M.O. is required to put the past history of the case on the first page, and the present condition of the patient, under a separate question, on the second. As regards the past history, he is specially requested to make a careful distinction between the unsupported statement of the patient and evidence which was either within the knowledge of the writer of the report or was supported by some documentary evidence. Many a man who has been in charge of a medical or surgical division will carry with him to the grave the grey hairs that he has developed in his attempts to explain these simple differences to M.O.s who have worked under him.

### Increase of Work.

We do not believe it will hurt the medical profession as a whole or any of us as individuals to do an increased amount of work; but we think that such increase would be better expended in other ways than the writing of notes. If such a system were introduced it would be bound to fail as soon as any severe crisis of work arose. To all of us there comes in our lives certain periods when we are working so hard that it is impossible to give that attention to our patients that we should wish. Under such circumstances something must go, and after the unnecessary little conversations about the weather, all clerical work will go first. Considering the subject from all points of view, we admit the advantages would be great if such a plan were carried out; but we very much doubt whether in the present state of human nature it ever would be efficiently done; and if it were not done efficiently we feel that it were better not done at all.

